# Adhesive Force Between Biconcave Red Blood Cell Membrane and Bulk Substrate

**DOI:** 10.3390/membranes15030089

**Published:** 2025-03-10

**Authors:** Weihua Mu

**Affiliations:** Wenzhou Key Laboratory of Biomaterials and Engineering, Wenzhou Institute, University of Chinese Academy of Sciences, Wenzhou 325000, China; muwh@ucas.ac.cn; Tel.: +86-10-8801-7527

**Keywords:** Ouyang–Helfrich equation, biconcave shape, red blood cells, van der Waals energy, Hamaker constant, adhesive forces

## Abstract

Adhesion between a red blood cell and substrates is essential to many biophysical processes and has significant implications for medical applications. This study derived a theoretical formula for the adhesive force between a red blood cell and a bulk substrate, incorporating the Hamaker constant to account for van der Waals interactions. The derivation is based on a biconcave shape of an RBC, described by the well-known Ouyang–Helfrich equation and its analytical solution developed by Ouyang. The theoretical predictions align with experimental observations and the empirical spherical model, revealing a $F\propto D^{-2.5}$ relationship for biconcave RBCs versus $F\propto D^{-2}$ for spheres. While the current study focuses on idealized geometries and static conditions, future work will extend these findings to more complex environmental conditions, such as dynamic flow and interactions with plasma proteins, thereby broadening the applicability of the model. This work bridges foundational research in cell membrane mechanics with practical applications in hemostatic materials, platelet adhesion, and biomaterials engineering. The findings provide insights for designing advanced biological sensors, surgical tools, and innovative medical materials with enhanced biocompatibility and performance.

## 1. Introduction

The study of cell adhesion is pivotal in biophysics, underpinning diverse biological processes, including cellular migration, tissue development, wound healing, and hemostasis. Among these, the adhesion between red blood cells and various substrates plays a vital role in both physiological and pathological contexts. The mechanics governing Red Blood Cell (RBC) adhesion are influenced by complex interactions at the nanoscale, such as van der Waals forces, electrostatic interactions, and membrane deformation. A comprehensive understanding of these interactions is crucial for elucidating the fundamental principles of membrane mechanics and for advancing applications in biomedical science [1].

The interaction between blood cells and medical devices is a critical factor in the performance and biocompatibility of these devices. Among various cell types, red blood cell (RBC) adhesion to biomaterial surfaces has garnered significant attention due to its implications for thrombogenicity and device functionality [2,3,4,5,6,7,8,9,10]. The surface properties of medical devices, such as wettability, surface charge, chemistry, and topography, play pivotal roles in determining the adsorption quantity and conformation of plasma proteins, which in turn influence platelet and RBC adhesion. Understanding these interactions is crucial for developing effective coatings that minimize thrombosis risk while maintaining optimal device performance. Additionally, computational modeling and experimental techniques have been employed to better understand and predict these complex interactions.

Recent advancements have focused on creating novel coatings for surgical instruments to reduce thrombogenicity. Polydopamine (PDA), for instance, has emerged as a promising coating material due to its excellent biocompatibility and ability to modify surface characteristics. Studies have shown that PDA coatings can significantly alter the adhesion behavior of blood components, including RBCs, by modifying surface energy and promoting favorable protein adsorption patterns. Moreover, computational models combined with experimental data provide insights into the underlying mechanisms of RBC adhesion, offering a pathway to optimize coating strategies.

In this context, Atomic Force Microscopy (AFM) has been instrumental in measuring adhesion forces at the single-cell level, providing detailed information on how different surface modifications affect cellular interactions. Building upon previous work, this study extends these investigations by conducting AFM measurements on single RBCs adhering to both silicone rubber and PDA-coated silicone rubber surfaces. Theoretical modeling and analysis of these adhesion forces was performed, achieving results that align well with experimental observations. This work builds on foundational studies by Wang et al. [11].

The adhesive forces between a biological cell and a substrate are intrinsically dependent on the cell’s shape, which is determined by the minimization of the free energy of the cell membrane. Among the components of this free energy, the bending energy is given as follows [12]:

(1) $$F_{b}=\frac{k_{c}}{2}\int {\left(2H+c_{0}\right)}^{2}dA+\bar{K}\int K\;dA,$$

where $k_{c}$ is the mean curvature modulus, $\bar{K}$ is the Gaussian curvature modulus, and $c_{0}$ is the spontaneous curvature. This bending energy functional, together with the surface tension and osmotic pressure terms, leads to the Ouyang–Helfrich equation for the equilibrium shape of an RBC membrane [13]:

(2) $$∆p-2\lambda H+k_{c}\left(2H+c_{0}\right)\left(2H^{2}-2K-c_{0}H\right)+2k_{c}\nabla^{2}H=0,$$

where *H* and *K* represent the mean and Gaussian curvatures, respectively, $\nabla^{2}$ is the Laplace–Beltrami operator, ∆p is the osmotic pressure difference, and λ is the surface tension coefficient acting as a Lagrange multiplier for surface area constraints. The Ouyang–Helfrich equation provides a relationship between the membrane’s geometry and its mechanical properties. Moreover, Ouyang derived an exact analytical solution to the Ouyang–Helfrich equation, offering precise descriptions of the RBC’s characteristic biconcave shape [13]. This solution is pivotal in studying adhesive forces, as it directly links the membrane’s mechanical properties to its interactions with substrates. The Ouyang–Helfrich equation, along with Ouyang’s exact solution, has significantly advanced our understanding of membrane mechanics and remains essential for both theoretical and experimental studies [1,13].

Experimental studies have provided valuable insights into the adhesive properties of RBC membranes under various conditions [14]. However, a robust theoretical model that integrates van der Waals interactions, as characterized by the Hamaker constant, with the membrane mechanics is still lacking. This work aims to address this gap by deriving a theoretical formula for the adhesive force between RBC membranes and substrates and comparing the results with experimental data [14].

Understanding the adhesive interactions of RBC membranes has broader implications beyond fundamental biophysics. The principles of cell adhesion are integral to the design of hemostatic materials, which mimic platelet adhesion for effective blood clotting [15], and to the development of biocompatible medical materials used in surgical equipment. Additionally, insights from this study could inform the design of biological sensors that rely on precise cell–substrate interaction measurements [15].

The Hamaker constant is a powerful tool for understanding adhesive forces, particularly in biological systems such as red blood cells [16]. Red blood cells often interact with each other and with vascular surfaces, where van der Waals forces play a significant role in adhesion and aggregation. Among the various methods used to quantify adhesive forces, the Hamaker constant provides a fundamental approach to describe the interaction energy between erythrocytes and other surfaces or particles. This constant is derived from Lifshitz theory [17], which accounts for the electromagnetic properties of the interacting materials, including their dielectric responses and refractive indices. By determining the Hamaker constant, researchers can predict the strength of van der Waals interactions, which are critical in determining the stability and adhesion behavior of red blood cells in different environments, such as plasma or under flow conditions. Thus, the Hamaker constant serves as a key parameter in bridging the gap between theoretical models and experimental observations of adhesive forces in biological systems.

This paper is structured as follows: I begin with a detailed theoretical derivation of the adhesive force, incorporating membrane mechanics and the Ouyang–Helfrich equation [18] and its analytical solutions. The derived formulas are then validated against experimental data from the literature. Finally, I discuss the implications of the findings for biophysics, medical applications, and future research directions in cell adhesion and membrane mechanics.

## 2. Modeling and Calculations

Previous models investigating RBC adhesion have often employed simplified geometries-such as spheres, cylinders, or flat surfaces to estimate adhesive forces via van der Waals (vdW) interactions, utilizing the Hamaker constant as an effective material parameter. For instance, the macroscopic theory of vdW dispersion forces has been applied to derive equations for the work of adhesion and contact angles in systems involving thin interfacial layers, with approximate expressions formulated in terms of Hamaker constants and surface energies. Additionally, studies have explored the calculation of Hamaker constants for organic materials, which are crucial for quantifying vdW forces at the nanoscale. These approaches, while foundational, often fall short in capturing the complex biconcave geometry of healthy RBCs under physiological conditions.

In the present work, I advance beyond these idealized models by considering the authentic biconcave shape of RBCs, as described by the Helfrich theory of membrane elasticity. This theory accounts for the bending energy of the membrane, providing a more accurate representation of RBC behavior in physiological environments. By incorporating the actual geometry and mechanical properties of RBCs, the model offers a more precise calculation of adhesive forces, which is essential for designing hemostatic materials and surgical instruments that interact with human blood. Understanding the true nature of RBC adhesion can inform the development of medical devices and materials that either promote or inhibit cell adhesion as required, thereby enhancing therapeutic efficacy and patient outcomes.

This refined modeling approach not only bridges the gap between theoretical predictions and biological reality but also provides practical insights for biomedical engineering applications. By aligning our models more closely with the physiological conditions of RBCs, we can better predict and control their interactions with various materials, leading to improved designs for medical devices and treatments. This work underscores the importance of accurate biological modeling in the advancement of medical technology and the development of effective therapeutic strategies.

## 3. Analytical Solution of Ouyang–Helfrich Equation for Axially Symmetric Red Blood Cell Membrane

**Figure 1:**
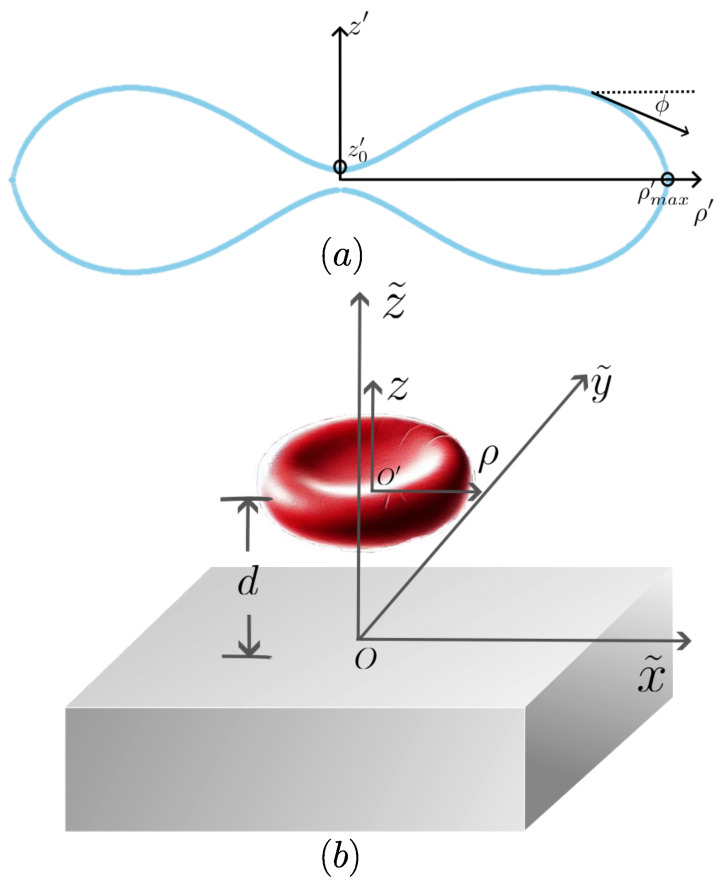
The shape of a red blood cell with the golden ratio on top of a bulk substrate. (**a**) The cross-section of a red blood cell membrane with a golden ratio character, passing through the symmetric axis $z^{\prime }$. The smallest thickness of the red blood cell $z_{0}^{\prime }$ and the maximum $\rho^{\prime }$, $\rho_{max}^{\prime }$, are marked at the curve of $z^{\prime }\left(\rho^{\prime }\right)$. (**b**) The coordinate systems for a red blood cell and a bulk substrate.

To solve the Ouyang–Helfrich equation, for an axially symmetric shape, the membrane is described by a contour in the ρ-*z* plane, where ρ is the radial distance from the axis of symmetry, and z(ρ) is the height of the membrane, as shown in Figure 1a. The shape can be parametrized by introducing the tangent angle ϕ, defined as the angle between the ρ-axis and the tangent vector of the curve z(ρ), with the relationship for the shape of the contour dz/dρ=tanϕ.

When the osmotic pressure and surface tension coefficient are negligible, Ouyang found an analytical exact solution as follows:

(3) $$\sin\phi =c_{0}\rho \ln\frac{\rho }{\rho_{\;\max}}-\frac{\rho }{\rho_{\;\max}},$$

where $\rho_{\max}$ denotes the maximum radial extent of the membrane, $\rho_{B}$ is the characteristic radius at which the tangent angle ϕ=0. The height z(ρ) of the membrane can be determined by integrating the tangent angle as follows:

$$z\left(\rho \right)=z_{0}+\int_{0}^{\rho }\tan\phi \left(\tilde{\rho }\right)d\tilde{\rho },$$

where $z_{0}$ is the central height at ρ=0.

The biconcave shape of an RBC reaches its maximum radial extent $\rho_{\;\max}$, where ϕ=−π/2. At this point, the tangent vector is perpendicular to the z′ axis. The conservation of the area of an RBC implies a natural length scale $R_{0}\equiv A_{\;\mathrm{RBC}}/\left(4\pi \right)$, which is the effective radius for a biconcave-shaped RBC; then, each quantity with a dimension of length *x* can be reduced to the corresponding dimensionless one by $x\prime \equiv x/R_{0}$, for a dimensionless parametric description of a surface for an RBC membrane.

## 4. The Beauty and Functionality of the Biconcave Red Blood Cell: Unveiling the Golden Ratio

The spontaneous curvature $c_{0}$ determines the cell’s overall geometry. For a healthy RBC, Ouyang and his co-researchers found the dimensionless parameter $c_{0}^{\prime }\equiv c_{0}R_{0}=-1.618$ corresponding to the negative golden ratio, representing an optimal configuration balancing deformability and mechanical stability [19]. The RBC membrane with the golden ratio suggests the radial extent $\rho_{\;\max}^{\prime }\equiv \rho_{\;\max}/R_{0}\approx 1.37$, and $\rho_{\;\max}/\rho_{B}\approx 1.6$, where $\rho_{B}$ is the radius at the point of maximal thickness, as shown in Figure 1. This result reflects the physiological necessity of RBCs to deform through narrow capillaries while maintaining sufficient structural integrity. The biconcave shape also minimizes surface area for a given volume, enhancing oxygen exchange efficiency while ensuring the RBC’s ability to pass the spleen’s rigorous filtration system, offering insights into their optimization through evolutionary selection.

Physiologically, the biconcave shape governed by these proportions ensures that RBCs efficiently traverse narrow capillaries while maintaining their ability to pass the spleen’s filtration system—a critical “physical fitness test” for cellular deformability. The shape minimizes surface area loss while maintaining sufficient elasticity, allowing RBCs to function optimally as oxygen carriers. This unique configuration also enhances laminar flow in large blood vessels, reducing turbulence and minimizing platelet activation, thereby preventing clot formation. These properties are crucial for maintaining efficient blood circulation and oxygen delivery [19].

From an applied mathematics perspective, the golden ratio in RBCs illustrates how natural forms arise as solutions to complex systems of differential equations [20,21]. The Ouyang–Helfrich equation provides a universal mathematical framework for understanding biological membranes. The presence of the golden ratio in the analytical solution underscores its ubiquity in natural optimization processes. Evolutionarily, this configuration may represent the culmination of selective pressures favoring an optimal balance between structural economy and functional adaptability. The conservation of this geometry across mammalian species further highlights its evolutionary significance.

It is possible to extend the implications of this discovery into fields such as biomimicry and synthetic biology. Artificial microfluidic devices inspired by RBCs could revolutionize drug delivery systems and blood substitutes by mimicking the deformability and efficiency of natural RBCs. Additionally, the role of van der Waals (vdW) forces in RBC adhesion, influenced by RBC thickness characterized by $|z_{\;\max}|$ and its dimensionless counterpart, shown in Figure 1a, has profound implications for understanding pathological conditions like malaria, where RBC rigidity is altered.

## 5. Results

To find the expression of the vdW energy between a biconcave RBC lying on a bulk substrate, we start with the parametric description of position vector $\vec{Y}\left(\rho \prime ,\theta \right)\equiv \left\{x,y,z\right\}$,

$$x=\rho \prime \cos\theta ,y=\rho \prime \sin\theta ,\;z\prime =\mp \int_{\rho \prime }^{\rho_{\;\max}^{\prime }}\;\tan\phi \left(\rho \prime \prime \right)\;d\rho \prime \prime ,$$

and the dimensionless volume element for the cell is obtained as $dV_{m}^{\prime }=-2\rho \prime \;d\rho \prime \;d\theta \;dz\prime \prime$, and $dV_{m}=dV_{m}^{\prime }\;R_{0}^{3}$, with

$$z\prime \prime \left(\rho \prime \right)\in \left[\int_{\rho \prime }^{\rho_{\;\max}^{\prime }}\;\tan\phi \left(\rho \prime \prime \right)\;d\rho \prime \prime ,\;-\int_{\rho \prime }^{\rho_{\;\max}^{\prime }}\;\tan\phi \left(\rho \prime \prime \right)\;d\rho \prime \prime \right],$$

which leads to the vdW energy, obtained as

(4) $$\begin{array}{ccc} & & U_{\;\mathrm{vdW}}=-C_{6}\rho_{s}\rho_{c}\;{\;\int \int \int \;}_{\;V_{s}}\;d\tilde{x}\prime d\tilde{y}\prime d\tilde{z}\prime \;\int_{0}^{\rho_{max}^{\prime }}\rho \prime \;d\rho \prime \;\int_{0}^{2\pi }\;d\theta \;\int dz\prime \;\\ & & \int_{-z^{\prime }}^{z^{\prime }}\;dz^{\prime \prime }\frac{\delta \left(z^{\prime }+\int_{\rho^{\prime }}^{\rho_{\;\max}^{\prime }}\;\tan\phi \left(\rho^{\prime \prime }\right)\;d\rho^{\prime \prime }\right)}{{\left[{\left({\tilde{x}}^{\prime }-\rho^{\prime }\cos\theta \right)}^{2}+{\left({\tilde{y}}^{\prime }-\rho^{\prime }\sin\theta \right)}^{2}+{\left({\tilde{z}}^{\prime }-z^{\prime \prime }-d^{\prime }\right)}^{2}\right]}^{3}}, \end{array}$$

which is reduced to

(5) $$U_{\;\mathrm{vdW}}=\frac{-A}{6}\;\int_{0}^{\rho_{\;\max}^{\prime }}\rho^{\prime }\;d\rho^{\prime }\;\left[\frac{1}{{\left(-z^{\prime }\left(\rho^{\prime }\right)+d^{\prime }\right)}^{2}}-\frac{1}{{\left(z^{\prime }\left(\rho^{\prime }\right)+d^{\prime }\right)}^{2}}\right],$$

with $z^{\prime }\left(\rho^{\prime }\right)=-\int_{\rho^{\prime }}^{\rho_{\;\max}^{\prime }}\;\tan\phi \left(\rho^{\prime \prime }\right)\;d\rho^{\prime \prime }$. Here, $\rho_{s}$ and $\rho_{s}$ are the density of a substrate and an averaged density of an RBC cell, respectively, and the Hamaker constant is $A\equiv \pi^{2}\rho_{s}\rho_{c}\;C_{6}$ [22].

In this study, Hamaker constants are used to characterize vdW interactions between RBC membranes and substrates. These constants were determined based on experimental data from the literature and theoretical analysis, referencing Israelachvili’s classical monograph. Specifically, We combined results from multiple experiments to establish a suitable numerical range for this study, typically falling within $10^{-21}$ to $10^{-19}\;\;J$, consistent with previously published studies on similar biological interfaces.

The Hamaker constant $A\equiv C_{6}\pi^{2}\rho_{1}\rho_{2}$ describes the van der Waals force, depending on material constants such as $C_{6}$ (the coefficient of the $1/R^{6}$ term in the Lennard–Jones potential) and the densities of two contacting bodies. In practical applications, it is generally used as an empirical parameter obtained from experiments. Under similar experimental conditions, different Hamaker constants corresponding to different material systems can be systematically studied.

Thus, the vdW energy is obtained as follows (see Appendix A for the details):

(6) $$U_{\;\mathrm{vdW}}\left(z^{\prime }\right)\approx -A{\left(\frac{\pi }{72}\right)}^{1/2}\frac{\rho_{B}^{\prime }}{{\left(d^{\prime }-\left|z_{\;\max}^{\prime }\right|\right)}^{3/2}},$$

For a perfect biconcave RBC with the golden ratio, $c_{0}^{\prime }=-1.618$, $\rho_{\;\max}^{\prime }\approx 1.37$, and $\rho_{B}^{\prime }\approx 0.87$, we obtain the following formula:

(7) $$U_{\;\mathrm{vdW},\;G-\mathrm{RBC}}\approx -0.18\frac{AR_{0}^{3/2}}{D^{3/2}}.$$

**Figure 2:**
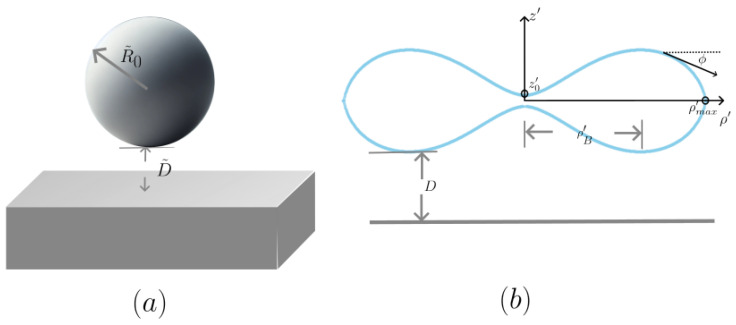
The distance between a spherical and biconcave red blood cell and the substrate. (**a**) A spherical cell with radius ${\tilde{R}}_{0}$; $\tilde{D}$ is the distance between the substrate plane and the lowest point of the cell. (**b**) A biconcave RBC with effective radius $R_{0}\equiv A_{\;\mathrm{RBC}}/\left(4\pi \right)$ ($A_{\;\mathrm{RBC}}$ is the area of the RBC, as mentioned in Section 3); *D* is the distance between the substrate plane and the lowest points of the RBC.

Here, $D\equiv D-|z_{\;\max}|$ is the minimal distance between the substrate surface and the biconcave RBC lying on the substrate, as shown in Figure 2b, which implies the following adhesive force behaviors:

(8) $$f_{\;\mathrm{adh},G-\mathrm{RBC}}=-\frac{dU_{\;\mathrm{vdW},G}}{dD}\approx -0.27\frac{AR_{0}^{3/2}}{D^{5/2}}\propto \frac{1}{D^{2.5}}.$$

It is well known that the adhesive force due to van der Waals interactions between a sphere and a bulk substrate takes the form

$$f_{\;\mathrm{adh},\mathrm{sph}}=-\frac{A{\tilde{R}}_{0}}{6{\tilde{D}}_{0}^{2}}\propto \frac{1}{{\tilde{D}}^{2}},$$

where ${\tilde{R}}_{0}$ is the radius of the sphere and ${\tilde{D}}_{0}$ is the minimum distance between the spherical surface and the substrate plane, as shown in Figure 2a. This formula is widely used to model the adhesive force of a real RBC in physiological conditions. This result closely resembles the form derived from the rough spherical model of an RBC.

## 6. Force–Distance Curves in Red Blood Cell Adhesion Measurements

Force–distance (F-D) curves are essential tools in atomic force microscopy (AFM) for quantifying the mechanical properties and adhesion behaviors of RBCs during their approach to and separation from substrates. These curves allow researchers to extract detailed nanomechanical properties of cells, providing insights into their viscoelasticity, elasticity, and adhesive interactions under physiological conditions. For example, Garcia et al. have developed methods to analyze F-D curves using bottom-effect corrections and power-law rheology models, transforming experimental data into viscoelastic parameters as functions of indentation frequency [23].

AFM studies on RBCs have been pivotal in exploring their mechanical responses, including topography, elasticity, and adhesion forces. Li et al. used AFM to investigate RBC mechanical properties, revealing valuable insights into their deformation and interaction with substrates [24]. These studies are crucial for understanding the behavior of RBCs under various physiological and pathological conditions. For instance, measuring the elasticity of RBCs can provide information on their ability to deform while traversing capillaries, a critical feature for oxygen delivery. Furthermore, adhesion measurements reveal how RBCs interact with endothelial cells, which is important in understanding diseases like malaria or sickle cell anemia.

Beyond AFM, other techniques such as dielectrophoretic (DEP) force measurements have also been employed to evaluate RBC mechanics. Jeon et al. demonstrated that DEP forces could assess RBC viability under oxidative stress, providing insights into the effects of storage periods on cell functionality [25]. By quantifying DEP forces using micro-electrode embedded chips and optical tweezers, their work highlights the importance of mechanical property assessments in medical diagnostics.

Despite these advances, many studies rely on simplified geometric models of RBCs, such as approximating the cell as a sphere or cylinder. While these models offer computational simplicity, they often fail to capture the intricate biconcave geometry of healthy RBCs. This limitation reduces their accuracy in describing adhesive forces and mechanical properties under realistic conditions. For instance, spherical models cannot accurately represent the non-uniform distribution of forces across the RBC membrane, which arises from its unique geometry.

In the present work, I address these limitations by incorporating the biconcave shape of RBCs into the calculations, as described by the Helfrich theory of membrane elasticity. This approach not only accounts for the cell’s actual geometry but also integrates its mechanical deformability and physiological functionality. By modeling the RBC as a biconcave disk, I provide a more precise estimation of adhesive forces during the approach and separation processes. The resulting force–distance curves capture the interplay between van der Waals forces, membrane elasticity, and cell-substrate interactions, enabling a comprehensive understanding of RBC adhesion.

This refined modeling approach has significant practical implications. The results can guide the design of hemostatic materials and surgical instruments that interact with human blood. By tailoring the surface properties of medical devices to optimize cell adhesion or prevent undesired interactions, this research contributes to advancing therapeutic efficacy and reducing complications in medical applications. The integration of realistic RBC geometry into adhesion studies bridges the gap between theoretical predictions and real-world biomedical applications, offering valuable insights for the development of blood-compatible biomaterials and diagnostic tools.

The Hamaker constants for the adhesive interaction between a biconcave RBC and a substrate were calibrated using unpublished AFM data [11]. Both constants are on the order of $10^{-20}\;\;J$ for the silicone rubber (SR) substrate and the polydopamine (PDA)-coated silicone rubber (SR-PDA) substrate. The ratio of $A_{\;\mathrm{SR}-\mathrm{PDA}}$ to $A_{\;\mathrm{SR}}$ is 1.7. Due to publication agreements, the raw data cannot be disclosed at this time.

While the primary analysis focuses on the power-law relationship between adhesion force and displacement based on experimental data, I acknowledge the importance of considering RBC deformation under varying mechanical stresses or substrate stiffness. During the stretching process, RBCs may deform; however, given that the AFM probe displacement does not exceed 20nm, which is significantly smaller compared to the characteristic lengths of biconcave discoid erythrocytes (micrometer scale), I did not account for RBC deformation in this work. Future work will aim to address these aspects to refine the model and better reflect real-world conditions.

## 7. Discussion

The derived formula for the adhesive force between the RBC membrane and a substrate provides a simplified yet effective approximation, which balances theoretical rigor and practical applicability. This result, grounded in the exact analytical solution of the Ouyang–Helfrich equation for membrane mechanics as first obtained by Ouyang, offers a foundation for understanding the interplay of physical forces driving adhesion phenomena in biological systems.

The formula explicitly incorporates the van der Waals interactions, parameterized by the Hamaker constant, and considers the elastic properties of the RBC membrane. Such a framework bridges the gap between classical membrane mechanics and modern experimental biophysics, offering a practical tool for experimental design and interpretation. By providing an approximate analytical expression, the derived model avoids the computational complexities of numerical simulations, making it more accessible for real-time experimental and industrial applications.

The Hamaker constant plays a crucial role in the study of biological and electromechanical systems, particularly in understanding van der Waals interactions where the distance between contact areas is on the order of nanometers. These interactions significantly affect processes such as adhesion, stability, and mechanical behavior. In the present study, for example, the Hamaker constant is used to model the adhesive force between a RBC membrane and a substrate, quantifying the van der Waals forces that govern the interaction between the cell membrane and the surface. Similarly, in a nanotube-based nanorelay, an electromechanical coupling device, the Hamaker constant helps model the adhesive force between a carbon nanotube (CNT) and an electrode, where van der Waals forces influence the working voltage, stability, and mechanical response of the system [26]. Understanding these forces is essential for designing and optimizing devices like nanoelectromechanical systems (NEMS) or biomaterials used in medical applications, where proper control over adhesive interactions is critical for functionality and reliability.

The Hamaker constant *A*, which characterizes the van der Waals interaction between two bodies, is often observed to be of similar order of magnitude for a wide range of materials. This universality arises from the relationship between the polarizability α, atomic or molecular volume *v*, and material density ρ. The coefficient *C* in the two-body potential is proportional to the square of the polarizability, and since α∝v and ρ∝1/v, the product of the densities $\rho_{1}\rho_{2}$ cancels the volume dependence. Consequently, the Hamaker constant *A* depends on the inverse of the volume product, leading to the observation that *A* typically falls within a similar range (around $10^{-20}$ to $10^{-19}\;\;J$) for many materials, despite differences in molecular or atomic structure. This results in a degree of universality in the Hamaker constant across various materials [27].

The consistency of the theoretical predictions with the empirical spherical model of RBC for reported experimental data underscores the validity of the proposed model. Deviations observed in specific scenarios may arise from factors such as heterogeneities in membrane composition, substrate irregularities, or non-ideal experimental conditions, which could serve as avenues for future refinement. This study highlights the potential for extending the model to include dynamic effects such as viscoelastic responses or transient forces during adhesion and detachment.

Applications of this work extend beyond fundamental biophysical research. In the context of hemostatic materials, the insights into RBC adhesion mechanisms can inform the design of bio-inspired materials that mimic platelet behavior for efficient clot formation. Similarly, the derived formula could aid in optimizing the adhesive properties of medical devices and surgical equipment for enhanced biocompatibility. Furthermore, the theoretical framework may contribute to the development of biological sensors that rely on precise cell-substrate interaction measurements.

Under physiological conditions, cell adhesion is influenced by a multitude of factors beyond the basic adhesive forces. These include fluid dynamics, which can significantly alter the mechanical stresses experienced by cells and thereby affect their adhesion behavior. Additionally, the roughness of the adhesive surface plays a crucial role in determining the efficacy and stability of cell attachment. Moreover, the involvement of membrane proteins associated with adhesion processes cannot be overlooked, as these molecules mediate specific interactions that are critical for stable cell anchoring. In future work, I aim to integrate these considerations by conducting experiments that account for the combined effects of fluid dynamics, surface characteristics, and membrane protein interactions. This comprehensive approach will allow for further elucidating the complex mechanisms underlying cell adhesion and potentially lead to more effective strategies for modulating adhesion in therapeutic contexts.

This study provides important theoretical support for biomedical fields, particularly in designing anti-adhesive coatings for medical devices. For example, understanding the adhesive forces between RBCs and various substrates can inform the development of advanced biological sensors, surgical tools, or hemostatic materials. I have expanded the discussion in the Section 1 to better illustrate these potential applications.

## 8. Conclusions

In summary, this study presents an approximate yet explicit formula for the adhesive force between a biconcave RBC and a bulk substrate, grounded in the exact solution of the Ouyang–Helfrich equation for RBC membranes. By integrating vdW interactions, parameterized by the Hamaker constant, with the elastic properties of the RBC membrane, the model provides a robust theoretical framework that aligns well with experimental observations. While the derived expression is approximate, it offers a practical and accessible tool for designing and interpreting experiments in cell adhesion mechanics, bridging the gap between theoretical membrane mechanics and experimental biophysics.

The developed theoretical model was calibrated based on unpublished AFM measurements performed by collaborators at Tsinghua University, China. While these experimental data have not yet been published, they provide critical support for the theoretical findings. Future work will aim to publish these experimental results and directly compare them with the theoretical predictions.

Three critical aspects currently constrain the model’s applicability: (1) static analysis neglects hemodynamic shear effects [6]; (2) substrate roughness is idealized as smooth; (3) protein-mediated adhesion, such as interactions involving CD47 (a membrane protein that plays a role in immune response and cell adhesion), is excluded. Future work will address these limitations through viscoelastic modeling and in vivo validation.

The broader implications of this work extend across biophysics, material science, and medical engineering. By elucidating the interplay of physical forces driving RBC adhesion, this study contributes to the development of advanced biomaterials, hemostatic agents, and biological sensors. Future research could expand the model to incorporate dynamic processes, substrate heterogeneities, or environmental factors, further enhancing its precision and applicability in both fundamental and applied contexts.

## Data Availability

Data supporting the theoretical models and calculations are available within the article. Unpublished AFM datasets referenced in this study are under the custodianship of the collaborating experimental team at Tsinghua University (Hongyu Zhang’s group).

## Abbreviations

The following abbreviations are used in this manuscript:

RBC	Red blood cell
vdW	van der Waals

## Appendix A

### Appendix A.1. Derivation of vdW Energy Between Biconcave RBC and Bulk Substrate Described by Equation (6)

We start with Equation (5) in the main text:

(A1) $$U_{\;\mathrm{vdW}}=\frac{-A}{6}\;\int_{0}^{\rho_{\;\max}^{\prime }}\rho^{\prime }\;d\rho^{\prime }\;\left[\frac{1}{{\left(-z^{\prime }\left(\rho^{\prime }\right)+d^{\prime }\right)}^{2}}-\frac{1}{{\left(z^{\prime }\left(\rho^{\prime }\right)+d^{\prime }\right)}^{2}}\right],$$

Obviously, it is known that the integrand is maximum at $\rho^{\prime }=\rho_{B}^{\prime }$, and we will consider the behavior of the integral

$$I_{1}\equiv \frac{1}{2}\int_{0}^{\rho_{\;\max}^{\prime \;2}}\;du\;\frac{1}{{\left(-\left|z^{\prime }\left(u\right)\right|+d^{\prime }\right)}^{2}}\equiv \frac{1}{2}\int_{0}^{\rho_{\;\max}^{\prime \;2}}\;du\;f\left(u\right),$$

with $u\equiv \rho^{\prime \;2}$ and $u_{0}\equiv \rho_{B}^{\prime \;2}$. We have $f\left(u_{0}\right)={\left(d^{\prime }-\left|z_{\;\max}\right|\right)}^{-2}$, $f^{\prime }\left(u_{0}\right)=0$, and

$$f^{\prime \prime }\left(u_{0}\right)=-\frac{1}{2}\frac{1}{{\left(\left|z_{\;\max}^{\prime }\right|-d^{\prime }\right)}^{3}}\frac{\phi^{\prime }\left(\rho_{B}^{\prime }\right)}{\rho_{B}^{\prime \;2}}=-\frac{1}{2}\frac{1}{{\left(d^{\prime }-\left|z_{\;\max}^{\prime }\right|\right)}^{3}}\frac{|c_{0}^{\prime }|}{\rho_{B}^{\prime \;2}}<0.$$

Thus, the integral $I_{1}$ is approximately

$$I_{1}\approx \frac{1}{2}\int_{-\infty }^{\infty }\;du\;\frac{1}{{\left(d^{\prime }-\left|z_{\;\max}^{\prime }\right|\right)}^{2}}\exp\left[-\frac{|c_{0}^{\prime }|{\left(u-u_{0}\right)}^{2}}{2\rho_{B}^{\prime \;2}\left(d^{\prime }-\left|z_{\;\max}^{\prime }\right|\right)}\right],$$

leading to the vdW energy having the form

$$U_{\;\mathrm{vdW}}\left(z^{\prime }\right)\approx -A{\left(\frac{\pi }{72}\right)}^{1/2}\frac{\rho_{B}^{\prime }}{{\left(d^{\prime }-\left|z_{\;\max}^{\prime }\right|\right)}^{3/2}}$$
